# Glutathione/pH-responsive nanosponges enhance strigolactone delivery to prostate cancer cells

**DOI:** 10.18632/oncotarget.26287

**Published:** 2018-11-09

**Authors:** Monica Argenziano, Chiara Lombardi, Benedetta Ferrara, Francesco Trotta, Fabrizio Caldera, Marco Blangetti, Hinanit Koltai, Yoram Kapulnik, Ronit Yarden, Luca Gigliotti, Umberto Dianzani, Chiara Dianzani, Cristina Prandi, Roberta Cavalli

**Affiliations:** ^1^ Department of Drug Science and Technology, University of Turin, Turin, Italy; ^2^ Department of Chemistry, University of Turin, Turin, Italy; ^3^ Agricultural Research Organization, Volcani Center, Rishon LeTsiyon, Israel; ^4^ Georgetown University Medical Center, Washington DC, USA; ^5^ Department of Health Sciences, Universita del Piemonte Orientale, Novara, Italy

**Keywords:** strigolactones, GSH/pH-responsive nanosponges, prostate cancer cells, controlled release, intracellular delivery

## Abstract

Strigolactones (SLs) are carotenoid-derived plant hormones that exhibit anti-cancer activities. We previously demonstrated that two SL analogues, MEB55 and ST362, inhibit the growth and survival of various cancer cell lines. However, these compounds have low aqueous solubility and stability at physiological pH. Here, we generated SL-loaded glutathione/pH-responsive nanosponges (GSH/pH-NS) to selectively deliver SLs to prostate cancer cells and enhance their therapeutic efficacy. The SLs were readily incorporated into the GSH/pH-NS. The drug loading efficiency was 13.9% for MEB55 and 15.4% for ST362, and the encapsulation efficiency was 88.7% and 96.5%, respectively. Kinetic analysis revealed that release of MEB55 and ST362 from the GSH/pH-NS was accelerated at acidic pH and in the presence of a high GSH concentration. Evaluation of the effects of MEB55- and ST362-loaded GSH/pH-NS on the growth of DU145 (high GSH) and PC-3 (low GSH) prostate cancer cells revealed that the GSH/pH-NS inhibited the proliferation of DU145 cells to a greater extent than free MEB55 or ST362 over a range of concentrations. These findings indicate GSH/pH-NS are efficient tools for controlled delivery of SLs to prostate cancer cells and may enhance the therapeutic efficacy of these compounds.

## INTRODUCTION

**Figure 1:**
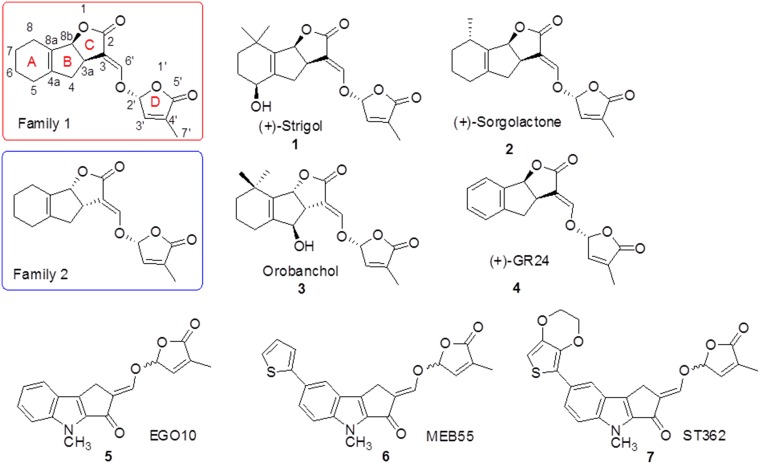
Chemical structures of several natural (Strigol, Sorgolactone, and Orobanchol) and synthetic SLs (GR24, EGO10, MEB55, and ST362)

Natural compounds with therapeutic activity can be exploited for cancer treatment because they are biocompatible and have well-characterized functions. Various plant-derived bioactive compounds have been shown to inhibit cancer cell growth and survival. Strigolactones (SLs) are carotenoid-derived plant hormones that are synthesized by plant roots and released into the rhizosphere [1–5]. SLs have a four-ring structure consisting of a tricyclic lactone (ABC rings) linked to a methyl butenolide (D ring) through an enol ether bridge (Figure 1). They are indispensable for the establishment of arbuscular mycorrhizae [6–9]. We previously demonstrated that two SL analogues, MEB55 and ST362 (Figure 1), could induce G2/M cell cycle arrest and apoptosis in a variety of human cancer cell lines *in vitro*. Additionally, they inhibited the growth of breast cancer stem cell-enriched mammospheres and human breast cancer xenograft tumors *in vivo* [10–11].

MEB55 was previously shown to have high anti-tumor efficacy and relatively low toxicity compared to conventional chemotherapeutics. Specifically, MEB55 disrupted the integrity of microtubule networks and inhibited the migration of highly invasive breast cancer cell lines [12]. More recently, SLs were found to promote genomic instability and cell death by inducing DNA damage and inhibiting DNA repair [13]. Although SL analogues are stable and readily available, they have several limitations including low aqueous solubility at basic pH [14]. The butenolide D-ring, which is the bioactiphore portion of the molecule, is easily hydrolyzed to generate an inactive compound. Therefore, more soluble and stable formulations are required for clinical applications.

Nanocarriers facilitate selective drug delivery and sustained release at target sites, thereby enhancing drug efficacy and reducing toxicity. The incorporation of drugs into nanocarriers can enhance their aqueous solubility and stability, because they protect them from the external environment. Nanocarriers can also alter the pharmacokinetics and biodistribution of the encapsulated drugs, and promote accumulation in tumor tissue owing to the enhanced permeation and retention (EPR) effect and cell internalization capability [15]. Nanocarrier-based drug delivery systems have therefore been developed to deliver therapeutics to tumors and enhance their effects [16–19]. For example, β-cyclodextrin (β-CD)- based nanosponges (NS) are solid, hypercrosslinked polymers with spherical morphologies that are a versatile platform for drug delivery [20–26].

Stimuli-responsive nanocarriers can allow sustained release of the encapsulated drugs in response to conditions in the microenvironment such as pH, enzyme concentrations, or redox gradients associated with various pathological states including neoplastic disease [27]. For example, glutathione (GSH)/pH-responsive NS (GSH/pH-NS) allow the controlled release of various drugs in response to the intracellular GSH concentration and pH [28–31]. The concentration of GSH is higher in tumor compared to normal tissue (0.5–10 mM vs. 2–20 μM) [32]. Additionally, the pH in the tumor microenvironment is more acidic than in normal tissue and blood (pH 6.2–6.9 vs. pH 7.4). NS are pH- and GSH-responsive owing to the presence of disulfide bridges and carboxyl groups in the GSH/pH-NS polymer matrix. Thus, a high intracellular GSH concentration can promote drug release from nanoparticles containing redox-sensitive chemical groups [28, 33–34].

GSH/pH-NS have been designed to deliver anticancer drugs to cells with high GSH levels. Importantly, doxorubicin-loaded GSH-targeted NS exhibited greater efficacy against cancer cells with high GSH content compared to free doxorubicin [35]. We hypothesized that GSH/pH-NS could enable the targeted delivery and controlled release of SL analogues (MEB55 and ST362) in prostate cancer cells thereby enhancing the therapeutic efficacy.

## RESULTS

**Figure 2:**
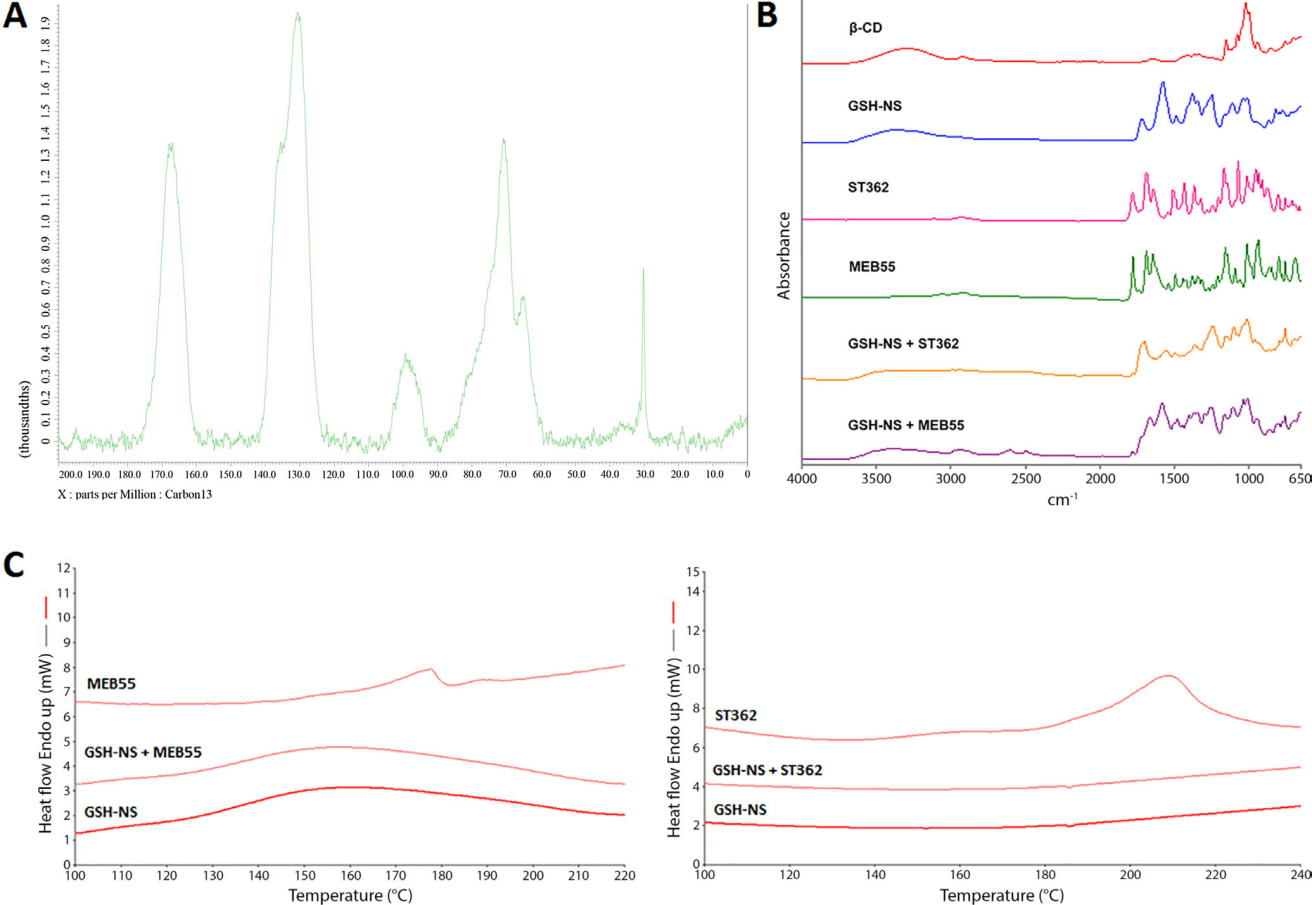
(**A**) ^13^C CP/MAS NMR (40–200 ppm) spectra of blank GSH/pH-NS, acquired with a spinning rate of 20 kHz at room temperature; (**B**) FTIR spectra of MEB55- and ST362-loaded GSH/pH-NS; (**C**) DSC thermograms of MB55- and ST362-loaded GSH/pH-NS.

We generated GSH/pH-NS in order to deliver two SL analogues (ST362 and MEB55) to prostate cancer cells. We first performed elemental analysis and solid-state nuclear magnetic resonance (SSNMR) spectroscopy to characterize unloaded (blank) GSH/pH-NS. The elemental analysis confirmed the presence of disulfide groups in the nanostructures. Additionally, CHNS analysis demonstrated carbon and hydrogen contents of 49.42% and 4.56%, respectively. The sulfur content was 0.74%, which was consistent with a previous report [28]. However, it was lower than the expected value of 0.97%, suggesting that 2-hydroxyethyl disulfide has less reactivity as crosslinking agent than pyromellitic dianhydride. The ^13^C cross-polarization/magic angle spinning (13C CP/MAS) SSNMR spectrum of the blank GSH/pH-NS is shown in Figure 2A. Several large signals at 168.2 ppm (carboxylic/ester groups), 130.9 ppm (aromatic C atoms), 100.9 ppm (O-C-O of the β-CDs, 71.6 ppm (C-O of the β-CDs and 2-hydroxyethyl disulfide) and lastly at 30.2 ppm (C-S belonging to 2-hydroxyethyl disulfide) were observed.

Then, a blank GSH-NS nanosuspension was prepared to obtain a nanoformulation for drug loading. A high pressure homogenization (HPH) step was performed to obtain NS with sizes in the nanometer range and a nearly homogeneous particle distribution. The average diameter of the blank GSH/pH-NS nanoformulation was approximately 200 nm.

The zeta potential of the blank GSH/pH-NS was approximately -30 mV, which was high enough to ensure the physical stability of the colloidal nanoformulations and avoid aggregation. The SLs were readily incorporated into the GSH/pH-NS. The drug loading capacity was 13.9% for MEB55 and 15.4% for ST362, and the encapsulation efficiency was 88.7% and 96.5%, respectively. The incorporation determined a huge increase of the SL apparent solubility. Indeed, the aqueous solubility of free MEB55 and ST362 was less than 0.2 μg/mL and 0.5 μg/mL, respectively, while an SL concentration of 1.5 mg/mL was achieved for both the compounds when incorporated into the GSH/pH-NS.

A comparison of the physical and chemical characteristics of the MEB55 and ST362-loaded GSH/pH-NS relative to the blank GSH/pH-NS is shown in Table 1. MEB55 and ST362 loading resulted in an approximately 8% increase in the size of the GSH/pH-NS and an approximately 15% decrease in the negative surface charge.

**Table 1:** Comparison of the physical and chemical characteristics of blank and SL-loaded GSH/pH-NS

Formulation	Average diameter ± SD (nm)	Polydispersity index	Zeta potential ± SD (mV)
Blank GSH/pH-NS	203.4 ± 12.3	0.20 ± 0.01	−31.5 ± 3.8
ST362-loaded GSH/pH-NS	219.8 ± 18.7	0.22 ± 0.02	−26.3 ± 2.5
MEB55-loaded GSH/pH-NS	217.3 ± 23.2	0.21 ± 0.02	−27.6 ± 2.3

**Figure 3:**
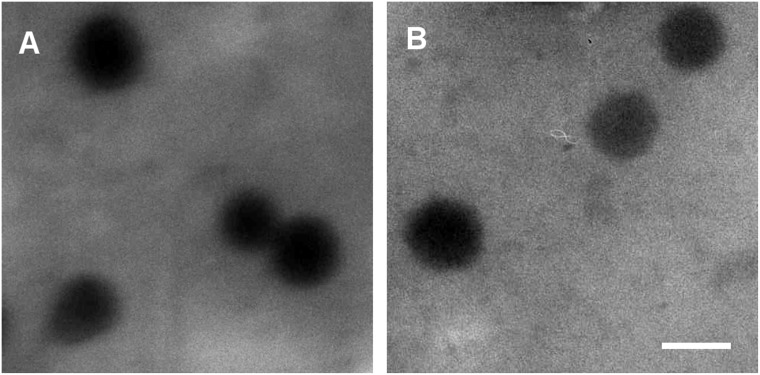
TEM images of (**A**) blank GSH/pH-NS and (**B**) SL-loaded GSH/pH-NS (scale bar = 200 nm).

We analyzed the size and morphology of the blank and loaded GSH/pH-NS using transmission electron microscopy (TEM). The GSH/pH-NS had spherical shapes and the sizes were in the nanoscale range (Figure 3A). No changes in morphology were observed after incorporation of the SLs into the GSH/pH-NS. Figure 3B reports TEM image of MEB55-loaded GSH/pH-NS. We next performed Fourier transform infrared spectroscopy (FTIR) and differential scanning calorimetry (DSC) to evaluate physical interactions between the SLs and the GSH/pH-NS. The FTIR spectra of the free SLs, SL-loaded GSH/pH-NS, and blank GSH/pH-NS are shown in Figure 2B. In addition to the fingerprint region, SLs exhibit intense absorption peaks in the 1800–1600 cm^-1^ range. The first peak (1780 cm^-1^) is due to the stretching of C = O bond in the D-ring of MEB55 and ST362, whereas the following bands derive from the absorption of C = O in the C-ring and C = C bonds adjacent to nitrogen and oxygen atoms. Less intense peaks appearing around 2900 cm^-1^ are associated with C-H stretching in CH, CH_2_ and CH_3_ groups. We observed changes and shifts in the MEB55 and ST362 peaks following incorporation into the GSH/pH-NS, reflecting the presence of physical interactions between the SLs and the GSH/pH-NS matrix. DSC thermograms confirmed that the SLs were incorporated into the GSH/pH-NS (Figure 2C). The endothermic melting peaks of MEB55 and ST362 (178°C and 210°C, respectively) were completely absent in the melting curves of the SL-loaded GSH/pH-NS, indicating the SLs were molecularly dispersed within the nanostructure of the GSH/pH-NS, and did not form crystals. Conversely, freeze-drying the free SLs resulted in the formation of a crystalline powder with a detectable melting peak at the fusion temperature of SLs (data not shown). These results were consistent with those of previous studies of other NS [36–39].

**Figure 4:**
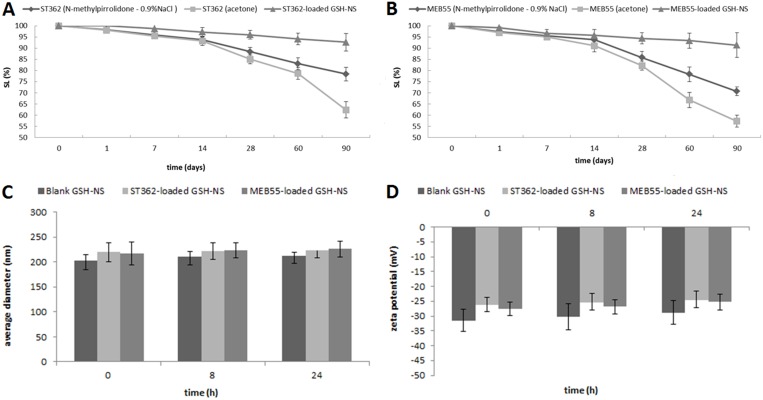
*In vitro* stability studies Chemical stability over time of (**A**) ST362 and (**B**) MEB55; (**C**) Average diameters and (**D**) Z-potentials of GSH/pH-NS formulations incubated in cell culture medium (*t* = 0, 8 and 24 hours). Each value represents the mean ± SD of 3 experiments.

Taking into account the stability issues of SL in solution, we analyzed the stability of the SLs dissolved in either acetone or N-methylpirrolidone-0.9% NaCl solution, and when loaded in the GSH/pH-NS. Encapsulation of the SLs in the GSH/pH-NS enhanced the chemical stability of the compounds compared to free SLs in the two solutions (Figure 4A, 4B). The concentrations of the SLs encapsulated in the GSH/pH-NS were 1.5 mg/mL for up to 3 months stored at 4°C. In contrast, a 22% decrease in the concentration was observed for free SLs in solution, which was indicative of reduced chemical stability. Moreover, we evaluated the physical stability of the GSH-NS nanoformulations. We monitored the average diameter and zeta potential of the GSH/pH-NS in cell culture medium and 0.9% NaCl solution over 24 hours. The GSH/pH-NS were stable in cell culture medium for up to 24 h, which was the timeframe used in our biological assays (Figure 4C, 4D).

**Figure 5:**
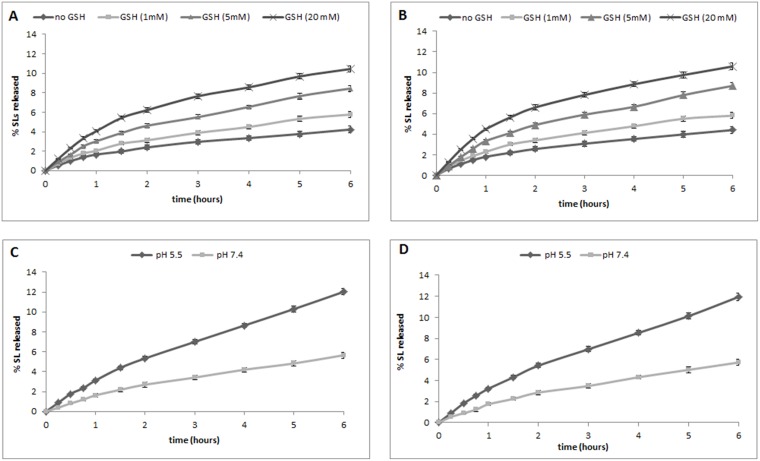
*In vitro* release kinetics of (**A**) ST362 and (**B**) MEB55 from GSH/pH-NS in the presence of increasing GSH concentrations (1, 5, and 20 mM), and of (**C**) ST362 and (**D**) MEB55 at pH 5.5 and 7.4 in the presence of 1 mM GSH.

We next investigated the GSH- and pH-dependent release of the SLs from the GSH/pH-NS. The *in vitro* release kinetics of ST362 and MEB55 from the GSH/pH-NS in the presence of increasing GSH concentrations (1, 5, and 20 mM) are shown in Figure 5A and 5B, respectively. We observed a slow and constant release profile with no initial burst effect for both the ST362- and MEB55-loaded GSH/pH-NS. SL-release from the GSH/pH-NS was dependent upon the GSH concentration in the receiving phase. The cumulative percent release was 2.5-fold higher in the presence of 20 mM GSH after 6 hours. We also analyzed the release kinetics of ST362 and MEB55 in the presence of 1 mM GSH at pH 5.5 and pH 7.4 (Figure 5C and 5D, respectively). Interestingly, we observed a pH/redox dual-responsive release profile for both SLs. The cumulative percent release of the SLs from the GSH/pH-NS was approximately 4% at pH 7.4 compared to 12% at pH 5.5 after 6 hours, indicating release was enhanced at acidic pH.

**Figure 6:**
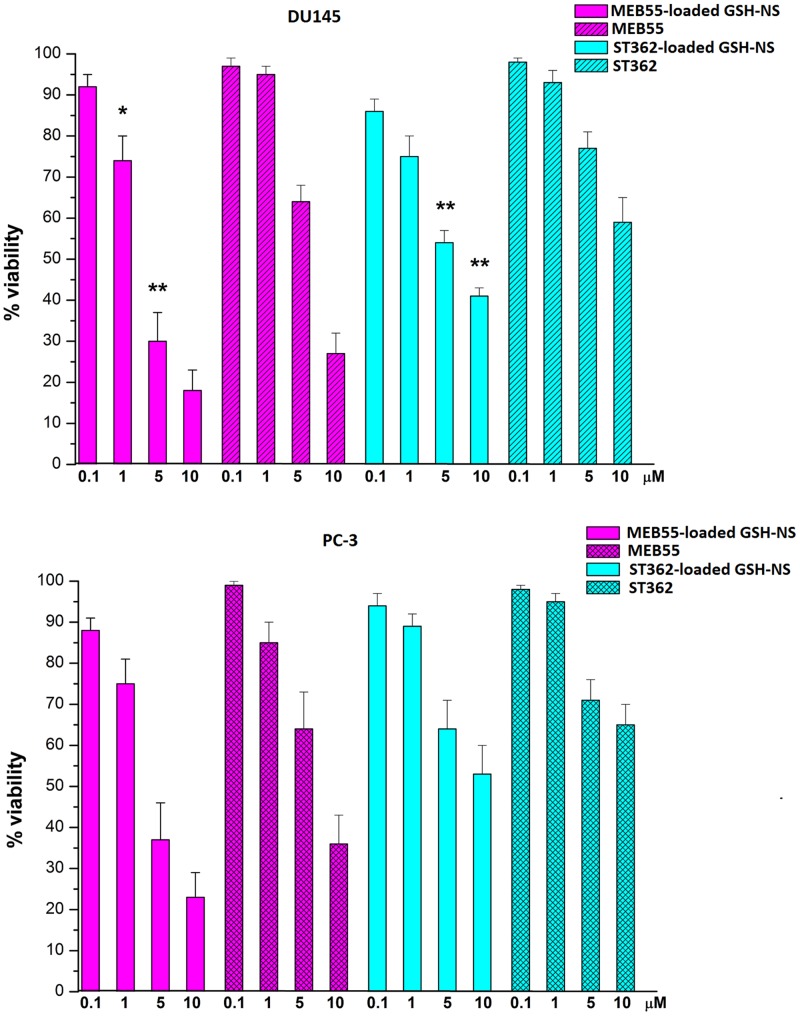
Analysis of cell viability 24 h after treatment of prostate cancer cells with free SLs or SL-loaded GSH/pH-NS The data are expressed as the percentage of viable cells relative to controls. ^*^p < 0.05.

We next evaluated the effects of MEB55- and ST362-loaded GSH/pH-NS on the growth of DU145 and PC-3 prostate cancer cells in culture. These cell lines displayed high and low GSH content, respectively [35]. Cell viability was analyzed after treatment of the cells for 24 h with a range of concentrations (0.1–10 μM) of free SLs or SL-loaded GSH/pH-NS using MTT assays. We found that MEB55 (both free and loaded in GSH/pH-NS) inhibited the proliferation of both cell types to a greater extent than ST362. Both SL-loaded GSH/pH-NS inhibited the proliferation of the DU145 cells (high GSH content) to a greater extent than the corresponding free drugs at all concentrations analyzed (Figure 6). In contrast, the SL-loaded GSH/pH-NS did not show statistically different effects on cell proliferation in PC-3 cells (low GSH content), compared to the corresponding free drugs, suggesting that the activity of the GSH/pH-NS is dependent upon the intracellular GSH content. The IC_50_ values for the free SLs and SL-loaded GSH/pH-NS are shown in Table 2.

**Table 2:** IC_50_ values for free SLs and SL-loaded GSH/pH-NS in PC-3 and DU145 prostate cancer cells

Formulation	PC-3	DU145
	IC_50_ (μM)	IC_50_ (μM)
MEB55	6.5 ± 0.8	6.4 ± 0.2
MEB55-loaded GSH/pH-NS	3.5 ± 1.2	2.4 ± 0.2^*^
ST362	19 ± 2.2	15 ± 0.5
ST362-loaded GSH/pH-NS	12 ± 1.8	6.0 ± 0.3^*^

^*^p < 0.05.

The blank GSH/pH-NS did not demonstrate any toxicity, even at the highest doses (data not shown), consistent with a previous study [35].

**Figure 7:**
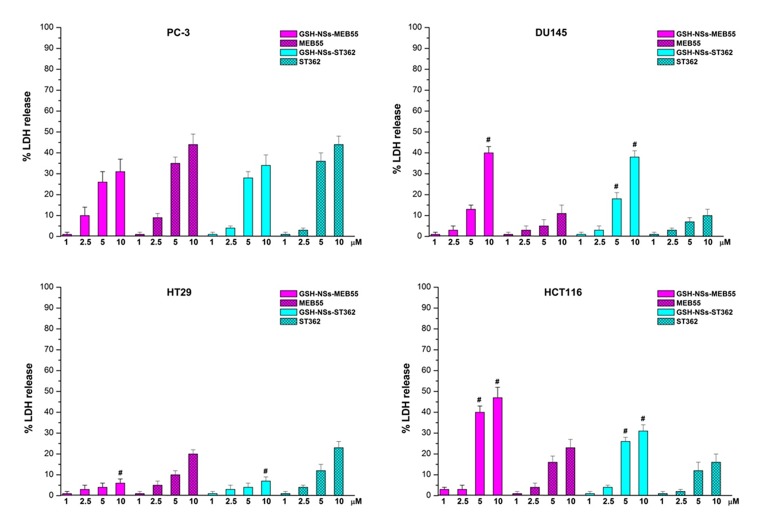
LDH release after treatment of the indicated cell lines with SLs or SL-loaded GSH/pH-NS for 24 h ^*^p ≤ 0.05.

To affirm the anticancer activity further, lactate dehydrogenase (LDH) assays were performed on DU145 and PC3 cells. Additionally, we analyzed other two colon cell lines, HCT116 and HT29 cells, which displayed high and low GSH concentrations, respectively [35], and compared them to normal NIH-3T3 fibroblasts. LDH release was observed in the culture medium of cells treated with SL formulations. The blank GSH/pH-NS did not show any toxicity, even at the highest concentrations (data not shown). The results indicated the efficacy of SL in inducing cell death by damaging the cell membrane. The percent LDH release was higher in DU-145 and HCT-116 cells treated with the SL-loaded GSH/pH-NS compared to free SL-treated cells (Figure 7).

The percentage of LDH release in HT29 cells (low GSH content) was higher after treatment with free SLs compared to SL-loaded GSH/pH-NS, indicating reduced compound delivery from the nanosponges in these cells after 24 h. Minimal SL release was observed in PC-3 cells (low GSH content). Finally, neither the free SLs nor the SL-loaded GSH/pH-NS were capable of inducing LDH release in normal NIH-3T3 cells.

We further confirmed the inhibitory effects of the SL-loaded GSH/pH-NS on cell proliferation using colony formation assays. This assay showed that SLs loaded in NS are able to reduce cell proliferation and that the antiproliferative effect was affected by the intracellular GSH content.

**Figure 8:**
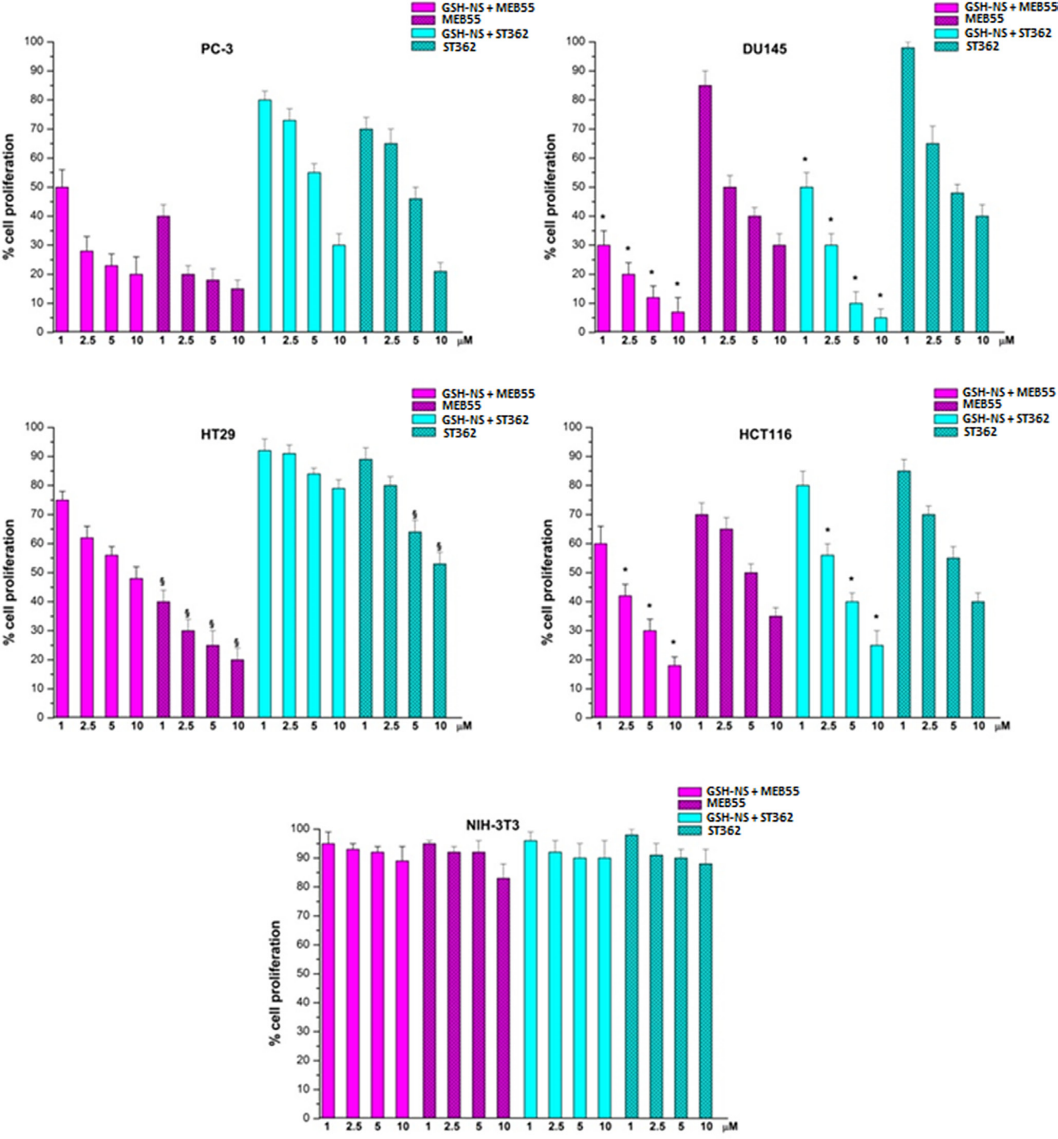
Colony formation assays of the indicated cell lines following treatment with SLs or SL-loaded GSH/pH-NS ^*^p ≤ 0.05 indicates a significant difference between cells treated with free SLs vs. SL-loaded GSH/pH-NS at the same concentrations. ^§^*p* ≤ 0.05 indicates a significant difference between cell lines treated with the same concentrations of SL-loaded GSH/pH-NS. Data are expressed as the percentage of cell proliferation.

Clonogenic assay results demonstrated that the inhibition of cell proliferation induced by SL-loaded GSH/pH-NS was in agreement with LDH release (Figures 7 and 8). Indeed, only cells with intact membranes and reproductive capacity were able to form colonies.

**Figure 9:**
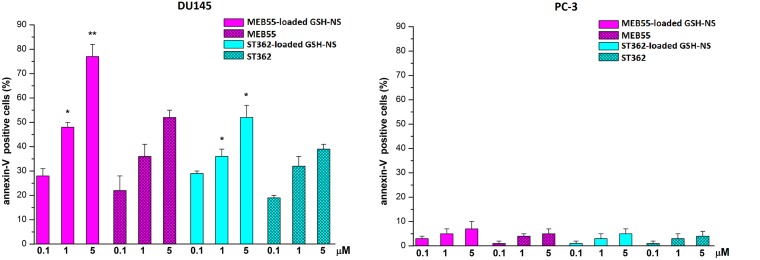
Levels of Annexin-V-positive cells after treatment for 24 h with either free SL or SL-loaded GSH/pH-NS Annexin-V-positive DU-145 (left panel) and PC-3 (right panel) cells. The results are expressed as the percentage of positive cells ^*^p < 0.05; ^**^p < 0.01.

We next investigated whether the inhibitory effects of the SL-loaded GSH/pH-NS on the growth of PC-3 and DU145 cells were due to apoptosis by staining the cells with Annexin V after treatment with free SLs or SL-loaded GSH/pH-NS (0.1–5 μM) for 24 h (Figure 9). The number of Annexin V-positive cells was higher among DU145 cells treated with the SL-loaded GSH/pH-NS compared to free SL treated cells (Figure 9). In contrast, there were few Annexin V-positive PC-3 cells following treatment with free SLs and SL-loaded GSH/pH-NS, which likely reflected reduced release of SLs in these cells. We observed no differences in cell death between DU145 and PC3 cells treated with free MEB55 or ST362. These data indicated that the release of the SLs from the GSH/pH-NS was stimulus-dependent.

**Figure 10:**
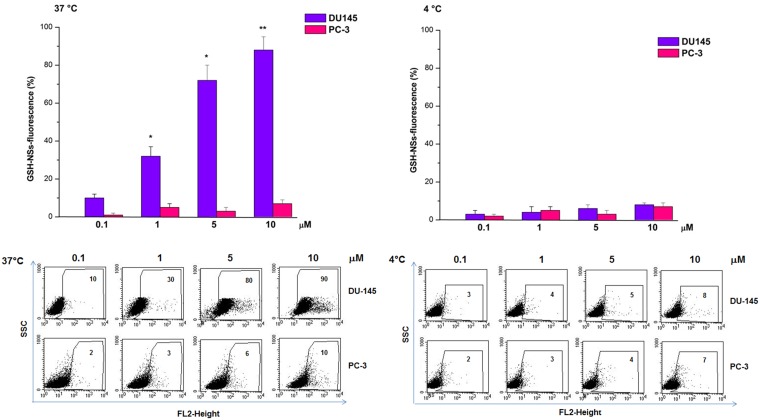
Flow cytometry analysis of the uptake of the GSH/pH-NS by prostate cancer cells DU-145 or PC-3 cells treated with the GSH/pH-NS at 37°C (left panel) or 4°C (right panel). The data are expressed as the mean ± SEM for four independent experiments (^**^p < 0.01, ^*^p < 0.05 compared to controls, paired *T*-test). Fluorescence activated cell sorting plots represent a single experiment.

**Figure 11:**
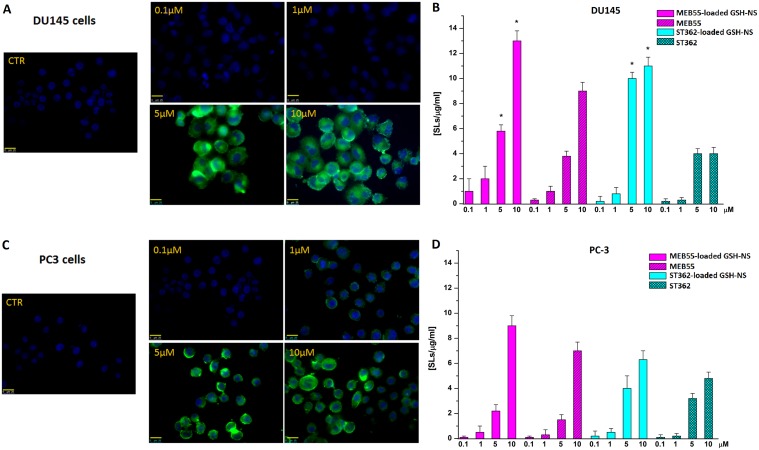
Fluorescence images (400X magnification) of the GSH/pH-NS (green) in DU-145 (**A**) and PC-3 (**C**) cells. Comparison between untreated cells (CTR) and cells treated with 0.1–10 μM GSH/pH-NS after 4 h; (**B**) Intracellular SL content in DU-145 (**B**) and PC-3 (**D**) cells 24 h after treatment with 0.1–10 μM free SLs or SL-loaded GSH/pH-NS.

We previously demonstrated that doxorubicin-loaded GSH/pH-NS were rapidly internalized by cancer cells, exploiting the intrinsic fluorescence of doxorubicin [35]. Here, GSH/pH-NS were loaded with a fluorescent marker, 6-coumarin, being an insoluble molecule in water [25]. Previously, we demonstrated that it was easily incorporated in the nanosponge matrix and was not released overtime. As a consequence, the internalization referred to the fluorescent nanoparticles. Cellular uptake of the fluorescently labelled GSH/pH-NS was analyzed in PC3 and DU145 cells after treatment for 4 h at either at 4°C or 37°C by flow cytometry (Figure 10). The percentage of fluorescent cells was dependent on the dose of the GSH/pH-NS and was higher after incubation at 37°C compared to 4°C in the DU145 cells. In contrast, the fluorescence was lower at both temperatures in PC-3 compared to DU145 cells (Figure 10). We evaluated the release kinetics of 6-coumarin from the GSH/pH-NS and observed a negligible release in the receiving phase after 24 h in the absence of GSH, as well in the presence of low GSH (data not shown). Fluorescence microscopy images demonstrating accumulation of the fluorescently labelled GSH/pH-NS in PC-3 and DU145 cells are shown in Figure 11.

We confirmed the intracellular accumulation of SLs in PC-3 and DU145 cells by measuring the SL concentration in cell lysates 24 h after incubation at 37°C with 0.1–10 μM free ST362 or MEB55 or the SL-loaded GSH/pH-NS using high performance liquid chromatography (HPLC) (Figure 11). Accumulation of the GSH/pH-NS was observed in all cells. The free drug content was higher in DU145 cells incubated with SL-loaded GSH/pH-NS than in cells incubated with the free SLs. At a concentration of 10 μM, the intracellular concentrations of MEB55 or ST362 were 1.52 and 2.68-fold higher, respectively, in cells treated with SL-loaded GSH/pH-NS relative to cells treated with free SLs. Thus, the GSH/pH-NS enhanced the accumulation of the SLs in prostate cancer cells.

## DISCUSSION

We developed novel GSH/pH-NS, responsive to pH and redox stimuli, to deliver two SL analogues (MEB55 and ST362) to prostate cancer cells with precise spatio-temporal control. The rationale of the nanoparticle design with multiple responsiveness was to provide ‘on demand’ targeted SL release as a function of the intracellular microenvironment. To our knowledge, this is the first work concerning the formulation of SLs in a nanocarrier, although Pollock *et al*. [11] previously demonstrated the efficacy of MEB55 and ST362 solubilized in acetone in primary prostate cancer cells.

We hypothesized that the incorporation of SLs into the GSH/pH-NS might circumvent issues with solubility and stability in aqueous solutions at physiological pH. Additionally, the low selectivity of a SL solution either in the therapeutic action or in the spatial distribution may lead to toxicity and low effectiveness. It is worth noting that the use of a drug within a nanodelivery system presents significant advantages versus normal cells. One of them concerns the capability to enhance the pharmacokinetics and biodistribution profiles, thereby improving drug efficacy and reducing side effects [40, 41]. GSH/pH-NS might modify the biodistribution and intracellular release kinetics of the incorporated SLs. Interestingly, previous *in vivo* experiments in animal models demonstrated that NS are able to modify the pharmacokinetics parameters of the loaded drugs [26, 38, 42]. Very recently, GSH-NS encapsulating doxorubicin exhibited a prolonged circulation time after intravenous administration to rats and were able to extravasate in tumor tissues exploiting the Enhanced Permeability and Retention (EPR) effect [35]. These findings motivate the encapsulation of SL in this type of nanoparticles.

We found that both MEB55 and ST362 were molecularly loaded into the GSH/pH-NS in a great extent. The incorporation avoided the compound crystallization as showed by DSC analysis. The SLs could be incorporated into the GSH/pH-NS as either inclusion or non-inclusion complexes, and they could interact with multiple sites in the nanosponge matrix, including the hydrophobic β-CD cavities and hydrophilic nanochannels of the polymeric network [20]. In addition, the slow *in vitro* release kinetics and the absence of an initial burst effect were suggestive of strong physical interactions between the SLs and the matrix of the GSH/pH-NS. The encapsulation of the SLs in the GSH/pH-NS protected them from chemical degradation and markedly increased the aqueous solubility. A thousand-fold enhancement of SL apparent solubility was reached. Indeed, due to compound loading, the solubility increased from 0.5 μM and 1.1 μM for MEB55 and ST362, respectively, to about 3 mM. These results are consistent with those of previous studies of other compounds [36–37]. The physical and chemical characteristics of nanoparticles (e.g. size, surface charge) are important to determine particle to cell interactions. They are crucial parameters in developing efficient nanomedicine with minimal toxicity. Specifically, the size of nanoparticles can impact cellular uptake [43]. The nanoscale range is advantageous for drug delivery because nanoparticles are more readily internalized by cells compared to microparticles [35, 44–45].

Particle size can also influence the pathway of cellular uptake. In the literature it is reported that spherical particles with size ≤ 200 nm enter the cell through clathrin-mediated cell uptake, while by increasing size to 500 nm, caveolae-mediated process is the predominant phenomenon in the cell internalization [46].

Based on these premises, the sizes of GSH/pH-NS were purposely tuned, using a HPH step in the preparation process, to obtain almost uniform small nanoparticles in the nanometer order of magnitude. We generated SL-loaded GSH/pH-NS with an average diameter of approximately 200 nm, which might facilitate rapid internalization into cancer cells and might exploit the EPR effect after administration [15].

In addition, surface charges are related to various biological performances of the nanoparticles [47]. The negative charge of the GSH/pH-NS can play a role in cellular uptake. It has been demonstrated that an increase in surface charge, either positive or negative, can enhance particle uptake compared to uncharged nanoparticles [43].

Stimuli responsive nanocarriers for anticancer drugs have attracted much research. They were designed to be inert during circulation, until they reach tumor tissue where they can receive the stimulus to release the drug. The intracellular SL release might be triggered by cellular environment. Various studies have shown that the intracellular pH is lower in cancer compared to normal cells [48]. Therefore, several pH-sensitive nanocarriers have been developed in order to deliver drugs under acidic conditions (i.e. pH 5.5 and 4.5, which correspond to the pH of endosomes and lysosomes, respectively) [49]. Moreover, the tumor reductive microenvironment, due to the higher GSH concentration than normal cells, is a feature for designing tumor-responsive nanotherapeutics. Particularly, dual redox and pH-responsive nanocarriers might have a great potential in the controlled release of a drug as a function of cellular environment. The presence of various targeting strategies in a drug-loaded nanomedicine is one of the novel approach to achieve combination therapy [50]. Dual redox/pH sensitive nanosponges are “smart” drug delivery systems designed to have high serum stability in physiological conditions and accumulate by EPR effect in the tumor microenvironment with peculiar biological parameters (i.e. low pH value and high GSH concentration). The dual stimuli-responsive GSH/pH-NS can provide a controlled drug release at a target site [51]. Interestingly, the *in vitro* release profiles from GSH/pH-NS were pH- and GSH-dependent. We found that the SLs were released from the GSH/pH-NS faster at pH 5.5 compared to pH 7.4 in the presence of high concentrations of GSH. These results are consistent with the chemical structures of the GSH/pH-NS, which contain free carboxylic groups in the polymer network and disulfide bridges that can be reduced in a reducing environment thereby favoring SL release. The free carboxylic group of the pyromellitic dianhydride, which was used as a cross-linker of β-CD, is only partly dissociated at pH 5.5. This results in reduced electrostatic interactions and enables release of the SLs in response to the intracellular pH and GSH concentration. The stimulus-dependent release of the SLs from the GSH/pH-NS was confirmed by *in vitro* experiments with prostate cancer cell lines.

Pollock *et al*. demonstrated that MEB55 and ST362 had the greatest cytotoxic effects in prostate and colon cancer cells compared to other SLs [11]. However, the SLs were evaluated in acetone in this study due to their low aqueous solubility, underscoring the need for more soluble carriers for preclinical and clinical applications. We observed a similar IC_50_ value for ST362 dissolved in N-methylpyrrolidone to the value reported by Pollock *et al*. The IC_50_ values of free MEB55 (6.4 and 6.5 μM for DU145 and PC-3 cells, respectively) were lower when it was dissolved in N-methylpyrrolidone, than those reported by Pollock *et al*. (33.5 and 27.3 μM for DU145 and PC-3 cells, respectively).

In this work, we investigated whether the SL-loaded GSH/pH-NS reduced the viability of prostate cancer cells *in vitro*. The MEB55-loaded GSH/pH-NS demonstrated greater inhibition of DU145 cell (high GSH content) viability in MTT assays compared to free MEB55. In contrast, they had a lower effect on the viability of PC-3 cells (low GSH content). LDH assays revealed that treatment with the GSH/pH-NS resulted in cell death, which was dependent on the intracellular GSH content. Treatment with free SLs also induced cell death in PC-3 cells, which was likely related to oxidative stress. Limited LDH release was observed in the other cell types 24 h after treatment. Annexin-V staining indicated that the DU145 cells underwent apoptotic cell death, as previously showed by Pollock *et al*. [11–12]. We also performed colony formation assays, which confirmed the dependence of the cellular responses on the intracellular GSH content.

Daga et al. [35] demonstrated that DU145, PC-3, HCT116, and HT29 cells had GSH contents of 12, 7, 14, and 4 μg per mg of protein, respectively. Additionally, they showed that the level of reactive oxygen species (ROS) was inversely correlated with the GSH content. PC-3 and HT29 cells displayed higher levels ROS than DU145 and HC116 cells, suggesting they had reduced antioxidant potential [35]. These data are consistent with the *in vitro* behavior of the SL-loaded GSH/pH-NS that we observed.

In summary, this work was focused on the design and development of a stimuli responsive nanomedicine for the delivery of SLs in prostate cancer cells. It is the proof of concept of the feasibility to incorporate SLs in a nanocarrier to improve effectiveness. We demonstrated that the incorporation of two SLs into GSH/pH-NS enhanced their cytotoxic effects on prostate cancer cells. Our data indicate that GSH/pH-NS are dual stimuli responsive nanocarrier that may favor the selectively controlled release of SLs in target cancer cells.

## MATERIALS AND METHODS

The β-CD was a gift from Roquette Italia (Cassano Spinola, Italy). All reagents were analytical grade and obtained from Sigma-Aldrich (St. Louis, MO, USA) unless otherwise specified. Cell culture reagents were purchased from Gibco/Invitrogen (Life Technologies, Paisley, UK) unless otherwise specified. The SL analogues were synthesized as previously described [14]. ST362 was a gift from StrigoLab Srl (Turin, Italy).

### Cell lines and culture

Cell lines were obtained from ATCC (Manassas, VA, USA). The cells were cultured as a monolayer in RPMI 1640 medium supplemented with 10% FCS, 100 U·mL^-1^ penicillin, and 100 μg·mL^-1^ streptomycin at 37°C in a 5% CO_2_ humidified atmosphere.

### Synthesis of the GSH/pH-NS

GSH/pH-NS were generated using the method developed by Trotta et al. [28]. Briefly, β-CD was reacted with pyromellitic dianhydride (crosslinking agent) and 2-hydroxyethyl disulfide (DHES) in dimethylsulfoxide (DMSO) in order to insert disulfide bridges in the polymer matrix. A total of 4.0 g (3.52 mmol) of anhydrous β-CD (desiccated in an oven at 100°C, up to constant weight) was dissolved in 16 mL of DMSO in a 100 mL round bottom flask. Once a clear solution was obtained, 0.400 g (2.59 mmol) of DHES and 4.0 mL (28.70 mmol) of triethylamine were added and the solution stirred for approximately 30 min. Finally, 11.01 g (48.96 mmol) of pyromellitic dianhydride was added to the reaction. The gelation point was reached after several minutes but the reaction was incubated for 24 h to reach completion. Once the reaction was complete, the monolith block was crushed with a mortar to obtain a coarse powder. The powder was rinsed with an excess of deionized water, filtered under vacuum, and then purified by means of Soxhlet extraction with acetone (for approximately 24 h). After air-drying, a white powder was collected and stored in a desiccator at room temperature.

### Elemental analysis

CHNS elemental analysis was performed to quantify the sulfur content in the GSH/pH-NS and compare it with the theoretical value. The CHNS analyses were performed in triplicate in a Thermo Electron Corporation Flash EA 1112 series CHNS-O Analyzer, using 2,5-bis (5-tert-butyl-benzoxazol-2-yl) thiophene (BBOT) as an external standard. Approximately 2.5 mg of each sample was placed in a tin capsule. An approximately equal quantity of V_2_O_5_ was then added as a catalyst.

### SSNMR spectroscopy

SSNMR spectra were acquired using a Jeol ECZR 600 instrument, operating at 600.17 and 150.91 MHz for ^1^H and ^13^C nuclei, respectively. Samples were packed into cylindrical zirconia rotors with a 3.2 mm optical density (OD) and a 60 μL volume. The ^13^C CP/MAS spectra were acquired at a spinning rate of 20 kHz using a ramp cross-polarization pulse sequence with a contact time of 3.5 ms, a 90° ^1^H pulse of 2.189 μs, (optimized) recycle delays of 2.26 s, and a total of 2,833 scans. A two-pulse phase modulation decoupling scheme was used to collect all spectra, with a radiofrequency field of 108.5 kHz. The chemical shift scale was calibrated relative to the methylene signal of glycine (43.7 ppm).

### Preparation of blank GSH/pH-NS

A top-down method was used to generate the GSH/pH-NS from a coarse powder. First, a weighted amount of the GSH/pH-NS was suspended in saline solution (0.9% w/v NaCl) at a concentration of 10 mg/mL while stirring at room temperature. The suspension was then dispersed using a high shear homogenizer (Ultraturrax^®^, IKA, Konigswinter, Germany) for 5 minutes at 24,000 rpm. The samples were subjected to HPH for 90 minutes at a back pressure of 500 bar using an EmulsiFlex C5 instrument (Avestin, Mannheim, Germany) to further reduce the sizes of the NS and obtain a homogenous distribution. The GSH/pH-NS were then purified by dialysis (Spectrapore cellulose membrane, MWCO: 12,000 Da) and stored at 4°C. A fraction of the GSH/pH-NS was freeze-dried using a Modulyo Freeze-Dryer (Edwards, Crawley, UK).

### Preparation of SL-loaded GSH/pH-NS

SL-loaded GSH/pH-NS were obtained by adding 1.5 mg/mL MEB55 or ST362 dissolved in 100 μL of N-methylpyrrolidone to an aqueous suspension of GSH/pH-NS at a concentration of 10 mg/mL. The mixture was then stirred at room temperature in the dark for 48 h. Unloaded SLs were separated from SL-loaded GSH/pH-NS by mild centrifugation. The SL-loaded GSH/pH-NS were then stored at 4°C until use. A select volume was freeze-dried to generate a solid powder. Weighted quantities of the SLs were dissolved in N-methylpyrrolidone and then diluted in 0.9% w/v NaCl for use as controls.

### Preparation of fluorescently labelled GSH/pH-NS

Fluorescently labelled GSH/pH-NS were obtained by adding 0.1 mg/mL 6-coumarin to an aqueous suspension of blank GSH/pH-NS at a concentration of 10 mg/mL as described above and then stirring the solution for 24 h at room temperature in the dark. Unloaded 6-coumarin was separated from fluorescent GSH/pH-NS by mild centrifugation.

### *In vitro* characterization of the GSH/pH-NS

The average diameter and polydispersity index of the GSH/pH-NS were determined using photon correlation spectroscopy (PCS). The zeta potential was determined from the electrophoretic mobility using a NanoBrook 90Plus instrument (Brookhaven Instruments Corporation, Brookhaven, NY, USA). Samples of each diluted solution of NS were placed in the electrophoretic cell and an electric field of approximately 15 V/cm applied. PCS was performed on GSH/pH-NS diluted in filtered distilled water with a scattering angle of 90° and at 25°C. The morphologies of the GSH/pH-NS were analyzed by TEM using a Philips CM10 instrument (Eindhoven, Netherlands). Aqueous suspensions of the GSH/pH-NS were sprayed onto Formvar-coated copper grids and air-dried prior to imaging.

### Thermal analysis

DSC was performed with a PerkinElmer DSC/7 (PerkinELmer, Shelton, CT, USA) equipped with a TAC 7/DX instrument controller. The instrument was calibrated using indium. The heating rate was 10°C/min and the temperature range was 25–250°C. Standard aluminum sample pans (Perkin-Elmer) were used to prepare samples. An empty pan was used as a reference standard. The analyses were performed in triplicate on 3 mg freeze-dried samples under a nitrogen purge.

### FTIR analysis

FTIR spectra of free SLs, blank GSH/pH-NS, and SL-loaded GSH/pH-NS were obtained in the 4000–650 cm^-1^ region using a Spectrum 100 FT-IR instrument (PerkinElmer). Data were analyzed using the Spectrum Software version 10.03.05 (PerkinElmer).

### Quantitative analysis of SL by HPLC

The concentrations of MEB55 and ST362 were quantified using an HPLC system consisting of a PerkinElmer PUMP 250B, equipped with a Flexar UV/Vis LC spectrophotometer detector (PerkinElmer, Waltham, MA, USA). We utilized a reversed phase Agilent TC C18 column (150 mm × 4.6 mm, pore size 5 μm; Agilent Technologies, Santa Clara, CA, USA). The mobile phase consisted of a mixture of acetonitrile and water (85:15 v/v), which was degassed and pumped through the column at a flow rate of 1 mL/min. The ultraviolet detector was set at 294 nm and 300 nm for MEB55 and ST362, respectively. The SL concentrations were calculated using a calibration curve and an external standard. A total of 1 mg of MBE55 or ST362 was placed in a volumetric flask and dissolved in acetonitrile to obtain a standard stock solution. This solution was then diluted in the mobile phase to generate the series of standard solutions. Linear calibration curves were obtained over a concentration range of 0.5−25 μg/mL. Both compounds had regression coefficients of 0.999.

### Analysis of the loading capacity of the GSH/pH-NS

Weighted quantities of freeze-dried MEB55- or ST362-loaded GSH/pH-NS were dispersed in 5 mL of acetonitrile. After sonication and centrifugation, the supernatants were analyzed by HPLC and the levels of MEB55 and ST362 in the GSH/pH-NS quantified. The loading capacity of the SL-loaded GSH/pH-NS was calculated using the following equation: [amount of SL/weight of NS] × 100.

### Analysis of the *in vitro* release kinetics of the SL-loaded GSH/pH-NS

*In vitro* assays of SL release from the GSH/pH-NS were performed using a multi-compartment rotating cell consisting of donor and receiving chambers separated by a cellulose membrane (Spectrapore, MWCO: 12,000 Da). A 1 mL volume of the SL-loaded GSH/pH-NS was placed in the donor chamber while 1 mL of phosphate-buffered saline (PBS) pH 7.4 containing 0.1% sodium dodecyl sulfate (SDS) to ensure drug solubility was placed in the receiving chamber. *In vitro* release studies were performed in the presence of increasing concentrations of GSH (1–20 mM) in the receiving compartment. The receiving phase was withdrawn at regular intervals and replaced with an equal volume of fresh solution in order to maintain sink conditions. The concentrations of the SLs were then analyzed by HPLC. The effects of pH on SL release from the GSH/pH-NS were analyzed using phosphate buffer containing 1 mM GSH at either pH 5.5 or 7.4 as the receiving phase.

### *In vitro* studies of the stability of the SL-loaded GSH/pH-NS

The physical stability of blank and SL-loaded GSH/pH-NS was analyzed in RPMI 1640 cell culture medium supplemented with 10% FCS or in 0.9% NaCl as a control. The SL-loaded GSH/pH-NS were incubated at 37°C and then the average diameter and Z-potential analyzed at 0, 8, and 24 h.

### *In vitro* evaluation of SL chemical stability over time

The chemical stability of MEB55 and ST362 was evaluated in acetone, N-methylpirrolidone-0.9% NaCl, and in the GSH/pH-NS by examining the SL concentration over time using HLPC as described above. The samples were stored at 4°C and analyzed at fixed time (0, 1, 7, 14, 28, 60, 90 days).

### Analysis of the intracellular concentrations of ST362 and MEB55

Prostate cancer cells (3 × 10^3^/well) were seeded in 96-well plates and incubated at 37°C in a 5% CO_2_ humidified atmosphere for 24 h. The cells were then incubated with increasing concentrations (0.1–10 μM) of MEB55, ST362, MEB55-loaded GSH/pH-NS, or ST362-loaded GSH/pH-NS for 24 h. Following the incubation, the cells were washed and lysed with a saturated solution of ammonium sulfate, and 100 μL of an acetonitrile: water mixture (70:30 v/v) added to the solution. The samples were then centrifuged at 4°C for 10 min and the supernatants collected and diluted with the mobile phase. The samples were then vortexed for 2 min, centrifuged, and the concentrations of the SLs in the supernatants quantified by HPLC as described above. The cellular uptake of ST362 and MEB55 was expressed as the intracellular SL concentration in μg/mL.

### Cell viability assays

MTT assays were performed to assess the viability of DU-145 and PC-3 prostate cancer cells. Cells (2 × 10^3^/well) were seeded into 96-well plates and incubated at 37°C in a 5% CO_2_ humidified environment for 24 h. The cells were then treated with increasing concentrations (0.1–10 μM) of MEB55, ST362, or the SL-loaded GSH/pH-NS. After 24 h, the absorbance was measured at 570 nm and the percentage of viable cells calculated using the manufacturer's protocol. Untreated control cells were normalized to 100%. Eight replicates were performed for each data point and a total of five independent experiments were performed.

### LDH assays of cytotoxicity

LDH leakage was estimated by measuring LDH activity in cell culture supernatants and cell lysates using a CytoTox 96 Non-Radioactive Cytotoxicity Assay Kit (Promega, Madison, WI, USA) according to the manufacturer's protocol [52]. DU-145, PC-3, HCT116, or HT29 cells (2 × 10^3^/well) were seeded into 96-well plates and incubated with increasing concentrations (0.1–5 μM) of MEB55, ST362, or the SL-loaded GSH/pH-NS for 24 h. Following the incubation, 50 μL of the Cytotox 96 Reagent was added to cell supernatants and the cells incubated at room temperature in the dark for 20 min. Culture medium was used as a control (background). Untreated control cell lysates were used as the Maximum LDH Release Control. The reaction was then terminated by the addition of 50 μL of the Stop Solution to each well. The absorbance was measured at 490 nm using a Victor Multilabel Plate Reader (PerkinElmer). LDH leakage was calculated using the following equation:

Percent cytotoxicity = 100 × [Experimental LDH Release – absorption of untreated control (OD490)]/[Maximum LDH Release – absorption of the untreated control (OD490)]. Four replicates were performed for each data point and a total of five independent experiments were performed.

### Colony formation assays

Cells (2 × 10^3^/well) were seeded into six-well plates. The following day, they were treated with increasing concentrations (0.1–5 μM) of MEB55, ST362, or the two SL-loaded GSH/pH-NS. The media was exchanged after 24 h and the cells cultured for additional 7 days in drug-free media. Following the incubation, the cells were fixed and stained with 80% crystal violet (Sigma-Aldrich) and 20% methanol. Colonies were then photographed and counted using a BioRad Gel Doc system (Bio-Rad Laboratories, Milan, Italy). The cells were then washed and 30% v/v acetic acid added to induce dissolution of the crystal violet. The absorbance was measured at 595 nm using a 96-well ELISA plate reader. A total of five independent experiments were performed.

### Annexin V staining

Cells (1.5 × 10^7^) were treated with increasing concentrations (0.1–10 μM) of SLs or SL-loaded GSH/pH-NS. After 24 h, the cells were stained with Annexin V using the Annexin-V-FLUOS Staining Kit (Becton Dickinson, Franklin Lakes, NJ, USA) and analyzed by flow cytometry. Dead cells displayed shrunken/hyper-granular morphologies and were Annexin-V-negative.

### *In vitro* studies of cellular uptake

Flow cytometry assays of the uptake of fluorescently labelled GSH/pH-NS by DU-145 and PC-3 cells were performed using a FacsCalibur flow cytometer (BD Biosciences, San Jose, CA, USA). Cells were seeded in 6-well plates (10^5^ cell/well) and incubated in the presence or absence of increasing concentrations (0.1–10 μM) of fluorescently labelled GSH/pH-NS at either 37°C or 4°C for 4 h. The cells were then washed twice with cold PBS to remove the unbound NS, trypsinized, and re-suspended in 500 μL of 1% paraformaldehyde. Live cells were analyzed by flow cytometry and the results expressed as the percentage of positive (fluorescent) cells. Uptake was confirmed by fluorescence microscopy. Cells were seeded in 24-well plates (5 × 10^5^ cell/well) on sterile coverslips and incubated in the presence and absence of increasing concentrations (0.1–10 μM) of the GSH/pH-NS at 37°C for 4 h. The cells were then rinsed three times with cold PBS and fixed with 4% paraformaldehyde for 30 min at 4°C. The nuclei were stained with DAPI (1 mg/mL; Sigma-Aldrich). The coverslips were then inverted and and mounted on glass slides. Images were acquired with a fluorescence microscope at 400X magnification (Leica DM5500 Microsystems, Milan, Italy) and analyzed using the LasX Software.

### Statistical analysis

Data are expressed as the mean ± standard error of the mean (SEM). Significant differences between experimental groups were detected by one-way ANOVA followed by Bonferroni correction using GraphPad InStat software (San Diego, CA, USA). A *p* value < 0.05 was considered statistically significant.

## Abbreviations

13C CP/MAS: 13C Cross-Polarization/Magic Angle Spinning
β-CD: β-cyclodextrin
BBOT: 2,5-bis(5-tert-butyl-benzoxazol-2-yl)thiophene
DAPI: 4′,6′-diamidino-2-phenylindole
DHES: 2-hydroxyethyl disulfide
DMSO: Dimethylsulfoxide
DSC: Differential scanning calorimetry
EPR: Enhanced permeability and retention
FTIR: Fourier transform infrared spectroscopy
GSH: Glutathione
GSH/pH-NS: Glutathione/pH-responsive nanosponges
HPH: High pressure homogenization
HPLC: High performance liquid chromatography
LDH: Lactate dehydrogenase
MTT: 2,3-bis[2-methoxy-4-nitro-5sulphophenyl]-2H-tetrazolium-5carboxanilide
OD: Optical density
PBS: Phosphate-buffered saline
PCS: Proton correlation spectroscopy
ROS: Reactive oxygen species
SDS: Sodium dodecyl sulfate
SL: Strigolactone
SSNMR: Solid-state nuclear magnetic resonance
TEM: Transmission electron microscopy.
